# SRPNet: stroke risk prediction based on two-level feature selection and deep fusion network

**DOI:** 10.3389/fphys.2024.1357123

**Published:** 2024-11-11

**Authors:** Daoliang Zhang, Na Yu, Xiaodan Yang, Yang De Marinis, Zhi-Ping Liu, Rui Gao

**Affiliations:** ^1^ School of Control Science and Engineering, Shandong University, Jinan, China; ^2^ Department of Rehabilitation Medicine, Affiliated Hospital of Jining Medical University, Jining, China; ^3^ Department of Clinical Sciences, Lund University, Malmö, Sweden

**Keywords:** stroke risk prediction, feature selection, deep fusion network, transformer, stroke risk factors

## Abstract

**Background:**

Stroke is one of the major chronic non-communicable diseases (NCDs) with high morbidity, disability and mortality. The key to preventing stroke lies in controlling risk factors. However, screening risk factors and quantifying stroke risk levels remain challenging.

**Methods:**

A novel prediction model for stroke risk based on two-level feature selection and deep fusion network (SRPNet) is proposed to solve the problem mentioned above. First, the two-level feature selection method is used to screen comprehensive features related to stroke risk, enabling accurate identification of significant risk factors while eliminating redundant information. Next, the deep fusion network integrating Transformer and fully connected neural network (FCN) is utilized to establish the risk prediction model SRPNet for stroke patients.

**Results:**

We evaluate the performance of the SRPNet using screening data from the China Stroke Data Center (CSDC), and further validate its effectiveness with census data on stroke collected in affiliated hospital of Jining Medical University. The experimental results demonstrate that the SRPNet model selects features closely related to stroke and achieves superior risk prediction performance over benchmark methods.

**Conclusions:**

SRPNet can rapidly identify high-quality stroke risk factors, improve the accuracy of stroke prediction, and provide a powerful tool for clinical diagnosis.

## 1 Introduction

Stroke is a global public health issue, ranking as the second leading cause of death and the third leading cause of disability worldwide (Owolabi et al., 2022). Moreover, the incidence of stroke is increasing in recent years, and the burden of stroke poses a huge challenge to low- and middle-income countries (Owolabi et al., 2021). However, the complexity, suddenness, and significant differences in clinical manifestations of stroke have brought great difficulties to treatment. It is widely acknowledged that stroke is preventable and controllable (Johnson et al., 2019). Therefore, active intervention on risk factors of stroke and accurate prediction of stroke risk through early screening can assist doctors and patients in implementing appropriate preventive and therapeutic measures, significantly reducing the harm caused by stroke.

So far, some studies employed traditional medical statistical methods to predict stroke risk (Wang et al., 2022; Abraham et al., 2021). These methods typically relied on a series of risk factors to construct mathematical models for calculating risk scores. However, these methods were time-consuming and labor-intensive, and ignored the complex nonlinear relationships and interactions among features, resulting in limited prediction performances (Obermeyer and Emanuel, 2016). With the rapid development of artificial intelligence, machine learning methods provide new solutions for stroke risk prediction. The machine learning methods can process complex screening data, and reveal patterns and associations hidden within large-scale data, thereby enhancing the accuracy of stroke risk prediction.

A better understanding of risk factors is critical for stroke diagnostic evaluation and treatment decision. In fact, controlling the risk factors (such as hypertension and diabetes) can reduce the risk of stroke. Qi et al. (2020) used multi-variable Cox regression analysis to obtain the features associated with the occurrence of stroke and its subtypes in China by introducing socioeconomic and other related factors. Abraham et al. (2019) employed elastic-net logistic regression to screen for genetic risk factors of stroke. Hunter and Kelleher (2023) used data from NHLBI Biologic Specimen and cardiac studies as risk factors, and studied the effect of age on stroke risk factors through a logistic regression algorithm. Maalouf et al. (2023) developed the regression model to find that negative emotions could increase stroke risk. Generally, stroke is a complex disease, and it is difficult to predict stroke risk via a single feature. However, having too many types of features may lead to redundant information and increase diagnostic costs. Furthermore, different risk factors contribute differently to stroke occurrence. More importantly, considering the association relationship among features is expected to be beneficial for the early stroke screening. Therefore, there is an urgent need to develop effective feature selection methods for predicting stroke risk.

Currently, numerous studies have been devoted to stroke risk prediction using machine learning techniques. For example, Li et al. (2019b) applied the Bayesian network model to estimate the incidence of stroke, revealing the relationship between combinations of multiple risk factors and stroke. Nwosu et al. (2019) analyzed the electronic health records of patients using neural networks, decision trees, and random forests to determine the impact of risk factors on stroke prediction. Arafa et al. (2022) developed a stroke risk prediction method for urban Japanese based on the Cox proportional hazards model, incorporating cardiovascular risk factors. Dritsas and Trigka (2022) designed an ensemble learning method for long-term stroke risk prediction. Liu et al. (2019) first adopted the random forest regression algorithm to impute missing data, and then used the deep neural network (DNN) to predict stroke on imbalanced physiological data. Although the above methods achieved promising results, the model structures they employed are relatively disconnected between features and algorithms, and the generalization ability of these models needs to be improved.

Here we propose a novel prediction model based on two-level feature selection and deep fusion network, termed SRPNet, for inferring stroke risk. In particular, two-level feature selection can comprehensively search for significant features related to stroke risk. We first apply multiple methods including Pearson correlation, chi-square test, Lasso and elastic net to select risk factors respectively, and combine the obtained risk factors as a candidate feature set. We then traverse all candidate risk factor combinations in the feature set by seven machine learning methods, such as support vector machine (SVM), k-nearest neighbor (KNN), decision tree (DT), gradient boosting decision tree (GBDT), random forest (RF), Gaussian Naive Bayes (GaussianNB) and AdaBoost, to identify the most important features associated with stroke. This enables evaluating the correlations between features and eliminating redundant information, providing reliable risk factors for stroke screening program. Next, the proposed deep fusion network integrates Transformer (Vaswani et al., 2017) and fully connected neural network (FCN) (Long et al., 2015) to establish a risk prediction model for stroke patients. This prediction model utilizes the attention mechanism of Transformer to explore hidden relationships among risk factors, and adopts FCN to better capture the nonlinear relationships among features. The experimental results indicate that SRPNet improves the accuracy and efficiency of stroke screening, and its performance is superior to existing benchmark methods. This work provides assistance for clinical diagnosis, and alleviates the burden of stroke.

## 2 Materials and methods

### 2.1 Datasets

The CSDC database covers 6 provinces, 41 hospitals and 12 population cohorts in China (Yu et al., 2016). The CSDC database facilitates stroke-related decision-making, research, and public health services through a comprehensive system. It collects and analyzes patient data, including risk factors, medical history, and sociodemographic information, ensuring that each subject has a unique record. A two-stage stratified cluster sampling method was employed during the data screening process (Li et al., 2019a). First, more than 200 screening areas were selected based on the local population size and the total number of counties. Then, urban communities and townships were used as the primary sampling units (PSUs) according to the geographical location and the recommendations from the local hospitals. In each PSU, all residents aged 40 and above were surveyed using cluster sampling during the initial screening period. Doctors assessed each patient’s condition, categorizing them as low risk, medium risk, high risk, transient ischemic attack (TIA), or stroke. The CSDC dataset comprises 862,244 middle-aged residents. Table 1 shows the detailed features of the CSDC dataset.

**TABLE 1:** Summary of specific features in the CSDC dataset.

Risk factors	Statistics	Abbreviation	Risk factors	Statistics	Abbreviation
Age group	54.48 ± 11.25	AG	Diabetes	49,674/812,570	Diabetes
Gender (male/female)	397,765/464,479	Gender	Lack of exercise	169,500/692,744	LE
Ethnic groups (minorities/majority)	2,716/859,528	EG	Overweight	148,834/713,410	Overweight
Occupation (mental/manual)	147,585/650,577	Occupation	Number of Marriages^c^	0.94 ± 0.23	NM
Education status^a^	1.79 ± 0.93	ES	Marital status	783,723/78,521	MS
Family history of stroke/hypertension/coronary heart disease^b^	60,320/801,924	FHS/HYP/CHD	Marriage_other	3,537/858,707	MO
History of stroke	16,862/845,382	HS	Provincial GDP	39.12 ± 15.88	PGDP
Hypertension	182,800/679,444	HYP	Province longitude	112.94 ± 6.61	PLO
Atrial fibrillation	23,445/838,799	AF	Province latitude	35.18 ± 2.96	PLA
Low-Density Lipoprotein Cholesterol	270,313/591,931	LDL-C	Province precipitation	721.27 ± 202.66	PP
Province’s highest temperature	26.83 ± 2.08	PHT	Province’s highest humidity	78.60 ± 4.67	PHH
Province’s lowest temperature	−0.09 ± 3.32	PLT	Province’s lowest humidity	55.36 ± 12.80	PLH
Smoking	155,982/706,262	Smoking	Category^d^	384,272/477,972	Category

^a^ The education status is divided into five levels, where 0 indicates illiteracy, 1 represents primary education, 2 represents secondary education, 3 represents higher education, and 4 represents postgraduate education.

^b^ The counts of subjects with and without family history of stroke/hypertension/coronary heart disease.

^c^ The number of marriages represents the number of times a subject has been married, with 0 for single, 1 for once married, and 2 for remarried.

^d^ The variable category represents the categories of random grouping.

The in-house data is sourced from the medical records of 49 patients at affiliated hospital of Jining Medical University in 2023. It includes 14 features such as gender, age group, ethnic groups, marital status, occupation, education level, hypertension, atrial fibrillation, smoking, hyperlipidemia, diabetes, overweight, and family history of stroke. Each patient has been diagnosed by a physician and classified as either having suffered a stroke or being in good health. The summary information for these two datasets is listed in Table 2.

**TABLE 2:** The detailed information of datasets.

Datasets	# of samples	# of features	Phenotypes of samples
CSDC	862,244	26	Low risk (612,819), Medium risk (124,103), High risk (85,155), TIA (23,305), Stroke (16,862)
In-house data	49	14	Health (24), Stroke (25)

### 2.2 Overview of SRPNet

**FIGURE 1:**
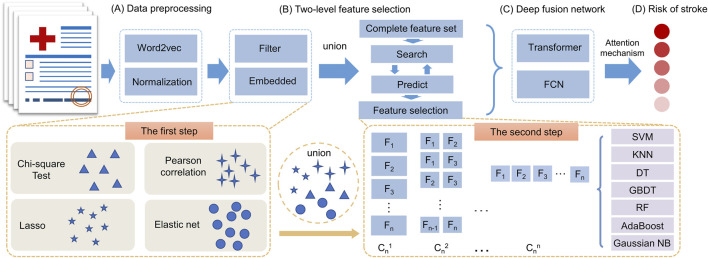
The entire framework of SRPNet. **(A)** Preprocess the input data. **(B)** Select significant features using the two-level feature selection. **(C)** Predict stroke risk based on the deep fusion network. **(D)** Output the stroke prediction results.

The SRPNet model mainly consists of two modules: two-level feature selection, and deep fusion network. The overall framework is illustrated in Figure 1. Since the dataset contains text information, the SRPNet firstly performs data preprocessing, which involves digitizing the textual information and normalizing the data. To eliminate low-correlation and redundant features, the two-level feature selection method is employed to identify comprehensive features associated with stroke. Finally, the deep fusion network, which adaptively fuse Transformer and FCN by attention mechanism, takes the obtained significant features as input to provide accurate stroke risk prediction results for stroke patients.

### 2.3 Data preprocessing

Based on the stroke risk researches (Tian et al., 2019; Guan et al., 2019), we used text information digitization to convert non-numeric features into numeric vectors suitable for machine learning or deep learning methods. Occupations are divided into mental workers and manual workers. For the marital status, we characterize it by whether the respondent is currently married and the number of marriage times. Based on the location information of the respondents, we convert it to the local climate, such as maximum temperature, minimum temperature, precipitation, humidity, etc. All of which are closely related to stroke. For the remaining features, we also use similar knowledge-based feature engineering for feature representation. Data normalization (Park et al., 2022) is used to scale data elements to the (0,1) interval, which helps improve the effectiveness and reliability of model training. The normalization formula is defined as follows Equation 1:

$$x^{\prime }=\frac{x-\min\left(x\right)}{\max\left(x\right)-\min\left(x\right)}.$$ (1)

### 2.4 Two-level feature selection

In this section, the two-level feature selection method that contains two-step feature selection processes will be introduced. The first step of feature selection involves four distinct methods, which are Pearson correlation, chi-square test, Lasso, and elastic net. The union of selected features from each method forms a set of candidate features. In the second step, based on the seven machine learning models, such as SVM, KNN, GBDT, RF, DT, AdaBoost and GaussianNB, we evaluate all possible combinations of candidate features via grid search. Each combination is scored based on its performance in the given models. It allows us to determine the optimal combination of features that are most predictive of stroke risk.

The two-step approach provides a rigorous feature selection process by multiple machine learning methods. The first step reduces the number of features based on statistical tests of relevance. The second step further refines the features by evaluating prediction performance in representative machine learning models. This ensures that the most informative and generalizable features have been selected for predicting stroke risk.

#### 2.4.1 The first step of feature selection

We employ four feature selection methods, including chi-square test, Pearson correlation, Lasso and elastic net, to assess the correlation between features and disease risk from different perspectives. The chi-square test and Pearson correlation prefer to filter out features, which have the advantage of high computational efficiency while not being prone to overfitting. However, their over-reliance on filter thresholds may overlook many important features. On the other hand, Lasso and elastic net are embedded feature selection methods that select salient features while accounting for feature correlations by calculating feature weights. Therefore, we combined the filter and embedded methods to comprehensively screen for the important features related to stroke risk factors. For details, we provide brief introductions to the chi-square test, Pearson correlation, Lasso, elastic net.

**Chi-square test** (Sharpe, 2015). The chi-square test is used to check the correlation of the independent variable with the dependent variable. We use the chi-square test to delete the features with small changes. The formula of chi-square test is described as Equation 2:

$$\chi^{2}=\sum \frac{{\left(A-E\right)}^{2}}{E},$$ (2)

where A is the observed value of the feature, and E is the expected value of the feature. The assumption of chi-square test is that features are independent. The larger result of the chi-square test means the higher correlation between features.

**Pearson correlation** (Cohen et al., 2009). We use Pearson correlation coefficient to measure the linear correlation between features and disease risk. When all the features have been scaled to (0,1), the most important feature should have the highest coefficient, and the irrelevant feature should have a coefficient whose value is close to zero. The Pearson correlation coefficient can be determined by Equation 3:

$$\rho_{x_{1},x_{2}}=\frac{\text{cov}\left(x_{1},x_{2}\right)}{\sigma x_{1}\sigma x_{2}}=\frac{E\left(x_{1}x_{2}\right)-E\left(x_{1}\right)E\left(x_{2}\right)}{\sqrt{E\left({x_{1}}^{2}\right)-E^{2}\left(x_{1}\right)}\sqrt{E\left({x_{2}}^{2}\right)-E^{2}\left(x_{2}\right)}},$$ (3)

where $x_{1}$ and $x_{2}$ represent the different feature, respectively. $\text{cov}\left(x_{1},x_{2}\right)$ denotes the covariance of $x_{1}$ and $x_{2}$ . $\sigma x_{1}$ denotes the standard deviation of $x_{1}$ , and $\sigma x_{2}$ denotes the standard deviation of $x_{2}$ .

**Lasso** (Nusinovici et al., 2020). Lasso built upon logistic regression analysis techniques, serves to select the most crucial features while reducing model complexity through the shrinkage of feature weights. Specifically, lasso introduces $L_{1}$ regularization into the loss function of a linear regression model, minimizing the mean squared error between predicted values and actual observations. The Lasso loss function is given by Equation 4:

$$\min_{\theta }\left\{\frac{1}{2n}\sum_{i=1}^{n}{\left(y_{i}-\theta_{0}-\sum_{j=1}^{p}x_{ij}\theta_{j}\right)}^{2}+\lambda \sum_{j=1}^{p}\left|\theta_{j}\right|\right\},$$ (4)

where $\theta =\left(\theta_{0},\theta_{1},...,\theta_{p}\right)$ denote coefficients that we need to compute. $y_{i}$ is the label that takes a value of 0 or 1. λ is a positive tuning parameter used to balance the loss term and penalty term. $x_{ij}$ represents the value of the j -th feature of the i -th sample.

**Elastic net** (Zhang et al., 2017). Since Lasso regression sometimes performs poorly in inter-correlated features, the elastic net was proposed to overcome this limitation. Elastic net regularization combines $L_{1}$ penalty with $L_{2}$ penalty together to select better relevant features simultaneously. The elastic net is defined as Equation 5:

$$\min_{\theta }\left\{\frac{1}{2n}\sum_{i=1}^{n}{\left(y_{i}-\theta_{0}-\sum_{j=1}^{p}x_{ij}\theta_{j}\right)}^{2}+\lambda \sum_{j=1}^{p}\left|\theta_{j}\right|+\left(1-\lambda \right)\sum_{j=1}^{p}\theta_{j}^{2}\right\},$$ (5)

where $\lambda \in \left[0,1\right]$ used to balance the $L_{1}$ penalty and $L_{2}$ penalty. The $L_{2}$ penalty of regularization term is defined as $\varphi \left(\theta ;\lambda \right)=\left(1-\lambda \right)\sum_{j=1}^{p}\theta_{j}^{2}$ , which is known as Ridge regression.

#### 2.4.2 The second step of feature selection

Although we have selected the important risk factors at the first step feature selection, the filter and embedded methods have the shortcomings of excessive threshold reliance and simply correlation consideration. To capture the deep correlation between features, we use seven machine learning methods to conduct the second step feature selection, which traverse all candidate features combinations based on the result of first step feature selection.

The candidate feature combinations consist of all possible permutations of the features selected during the process of feature selection. Assume there are n features that are selected. Then totally there are $2^{n}-1$ candidate feature combinations. Next, we traverse all candidate combinations using seven machine learning methods and select the optimal feature combination based on the classification performance of these seven different classifiers. As we know, machine learning methods are based on specific theoretical assumptions. Therefore, employing different machine learning methods can increase the diversity of feature selection. Brief introductions of the seven machine learning methods are presented in Table 3. These algorithms have their own advantages and disadvantages, allowing us to thoroughly consider different scenarios in the feature selection process.

**TABLE 3:** The overview of seven machine learning methods.

Methods	Theory	Advantages	Disadvantages
SVM (Noble, 2006)	Find the optimal hyperplane	Handling the interaction of nonlinear features	Difficulty in selecting the kernel function
KNN (Cunningham and Delany, 2021)	Find the k nearest neighbors	No assumptions, insensitive to outliers	Cannot handle imbalanced data
DT (Al Snousy et al., 2011)	Divides feature subspaces	Handle Boolean and numeric data simultaneously	Prone to overfitting
GBDT (Zhou et al., 2020)	Iteratively train decision trees	Strong interpretability	Difficulty tuning parameters
RF (Cutler et al., 2012)	Integrate several DTs	Strong generalization ability	Poor interpretability
AdaBoost (Schapire, 2013)	Integrated learning strategy	Prevent overfitting	Sensitive to outlier
GaussianNB (Liu et al., 2023)	Based on independence assumption	No need to tune parameters	Not suitable for high-dimensional data

### 2.5 Deep fusion network

**FIGURE 2:**
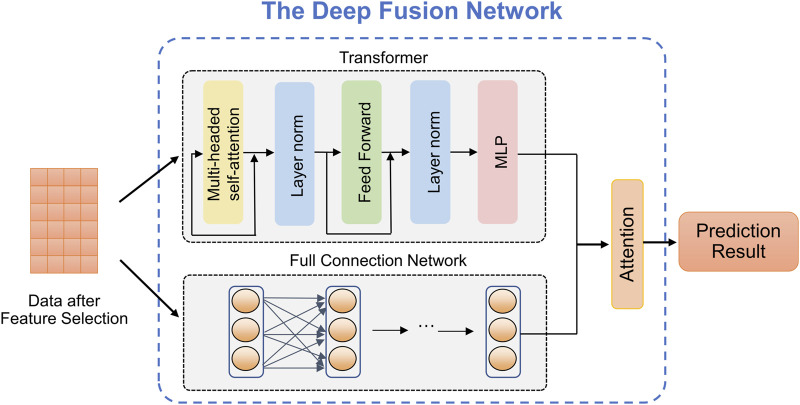
The structure of the proposed deep fusion network.

The complexity and diversity of stroke data require predicting stroke risk from multiple perspectives to enhance model robustness. Common predictive models, such as the Transformer, exhibit complex structures and excel at adapting to high-dimensional data, thus improving prediction performance. However, it often suffers from overfitting issues when dealing with small-scale datasets. In contrast, the FCN model has a simple structure and fast training speed, yielding exceptional performance on small-scale datasets. We utilize the advantages of both above predictive models and propose a deep fusion network method that can provide accurate the stroke risk prediction. As shown in Figure 2, deep fusion network integrates the Transform and the FCN, in which the dependencies between stroke risk factors are captured by the attention mechanism of the Transformer, and the complex nonlinear relationship is fitted by deep network structure of the FCN.

**Transformer** (Vaswani et al., 2017). Due to the powerful representation ability, Transformer can realize the outstanding performance in prediction tasks which is based on the self-attention mechanism. As observed in Figure 2, given an input $X\in {\mathbb{R}}^{n\times c}$ , where n represents the number of patients (or patches) and c represents the embedded feature dimension for every patient. The self-attention mechanism can be defined as Equation 6:

$$Attention\left(Q,K,V\right)=\text{softmax}\left(\frac{QK^{T}}{\sqrt{d_{k}}}\right)V,$$ (6)

where $d_{k}$ is the input matrix embedding dimension. The matrix Q , K , V can be computed by the input matrix and linear transformation matrix $W^{Q}$ , $W^{K}$ , $W^{V}$ , respectively. Then, we can get the value of Q , K , and V by computing $Q=XW^{Q}$ , $K=XW^{K}$ , and $V=XW^{V}$ , where $W^{Q}\in {\mathbb{R}}^{c\times q}$ , $W^{K}\in {\mathbb{R}}^{c\times q}$ , $W^{V}\in {\mathbb{R}}^{c\times q}$ , q denotes the linear mapping dimension.

**Fully connected neural network.** The FCN, also known as a Multilayer Perceptron (MLP), is a widely used artificial neural network structure in medical data analysis. It offers the advantages of fast training speed and robust modeling capabilities as the network depth increases. The stroke risk prediction model we designed includes one input layer, one hidden layer and one output layer. The calculation formula of each layer of network is defined by Equation 7:

$$y=\sigma \left(Wx+b\right),$$ (7)

where σ denotes the ReLU activation function. x is the input of the neuronal node, y is the output of the neuronal node. W and b denote weight and bias, respectively, which are learnable parameters.

**Attention mechanism.** In the stroke risk prediction task, the Transformer and the FCN extract clinical features at different levels and make distinct contributions to the prediction. Therefore, we introduce an attention mechanism to adaptively learn the importance of latent embeddings. Specifically, for the feature $H_{t}$ extracted by Transformer, we apply a non-linear transformation and employ the shared attention vector $W_{t}$ to obtain the attention coefficient $a_{t}$ , namely, Equation 8:

$$a_{t}=\text{softmax}\left(W_{t}\cdot \sigma \left(WH_{t}+b\right)\right),$$ (8)

where σ denotes the tanh activation, W denotes a trainable weight matrix, and b denotes a bias vector. Similarly, we can calculate attention coefficients $a_{c}$ for the features $H_{c}$ extracted by the FCN. We combine these embeddings to obtain the final embedding H , Equation 9:

$$H=L\left(a_{t}\cdot H_{t}+a_{c}\cdot H_{c}\right),$$ (9)

where L denotes the single linear layer.

### 2.6 Evaluation metrics

Here we employ four evaluation metrics to assess the predictive performance of the model, including micro precision, micro F1-score, macro precision, and Cohen’s Kappa coefficient (Younas et al., 2023). The definitions of these metrics are given as follows.

The micro average approach amalgamates performance measures across all samples. Specifically, for each class $g_{i}$ within the set $G=\left\{1,...,K\right\}$ , where K denotes the total number of classes, a dedicated confusion matrix is constructed. In this context, the i -th matrix designates the $g_{i}$ class as the positive class, while considering the remaining classes $g_{j}$ with j≠i as the negative classes. The micro precision and micro F1-score are computed by Equations 10 and 11:

$$P_{micro}=\frac{\sum_{i=1}^{\left|G\right|}TP_{i}}{\sum_{i=1}^{\left|G\right|}TP_{i}+FP_{i}},$$ (10)

$$F1_{micro}=\frac{2\sum_{i=1}^{\left|G\right|}TP_{i}}{2\sum_{i=1}^{\left|G\right|}TP_{i}+\sum_{i=1}^{\left|G\right|}FP_{i}+\sum_{i=1}^{\left|G\right|}FN_{i}},$$ (11)

where TP represents the number of positive samples correctly predicted to be positive samples, FP represents the number of negative samples incorrectly predicted to be positive samples, FN represents the number of positive samples incorrectly predicted to be negative samples.

Micro average tends to provide misleading results in the case of imbalanced data, as it doesn’t take the predictive performance of each specific class into account. In contrast, macro average computes averages through the individual performance of each class. The macro precision is defined as Equation 12:

$$P_{macro}=\frac{1}{\left|G\right|}\sum_{i=1}^{\left|G\right|}\frac{TP_{i}}{TP_{i}+FP_{i}}.$$ (12)

Cohen’s Kappa Coefficient is employed for assessing performance in situations of imbalanced class distribution, which is denoted by Equation 13:

$$kappa\left(k\right)=\frac{p_{o}-p_{e}}{1-p_{e}},$$ (13)

where $p_{o}$ denotes the overall model accuracy, and $p_{e}$ denotes the agreement expected by chance between the model’s predictions and the actual class values (McHugh, 2012).

### 2.7 Implementation details

The stroke risk prediction model was built and trained using the PyTorch. Experiments were conducted on a PC with Intel(R) Xeon(R) Gold 6258R CPU @ 2.70 GHz and NVIDIA QuADro GV100 GPU. We trained the model with the Adam optimizer (Kingma and Ba, 2014) with default parameters and a fixed learning rate of 0.001. And we randomly select 80% of the samples from whole dataset for training, and the remaining 20% for testing. The maximum number of epochs employed for training is 100. The datasets and source codes are publicly available on GitHub: https://github.com/zhangdaoliang/SRPNet.

## 3 Results and discussion

### 3.1 Two-level feature selection results

**FIGURE 3:**
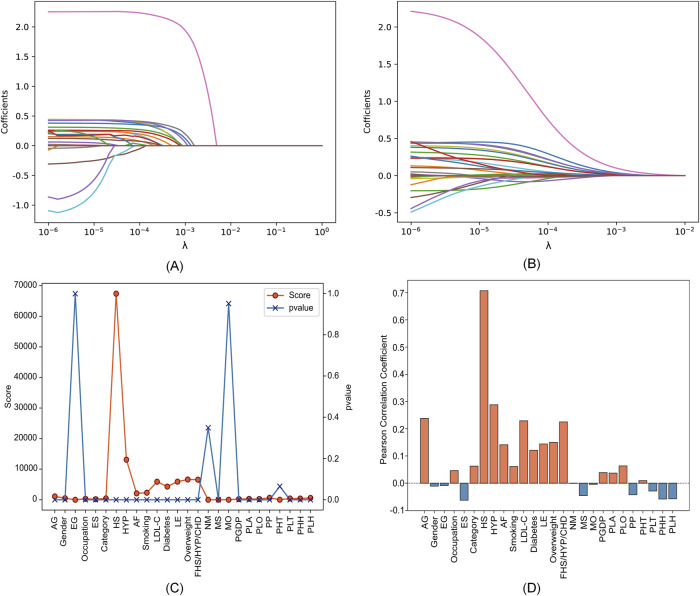
Feature selection results with different parameters in four feature selection methods. **(A)** Lasso. **(B)** Elastic net. **(C)** Chi-square test. **(D)** Pearson correlation.

We utilized a dataset from the CSDC database, consisting of 862,244 samples, with each sample originally having 26 distinct features. The proposed two-level feature selection method was used to screen out significant stroke features, which has a positive effect on improving the performance of the prediction model. In the first step of feature selection, we employed Lasso, elastic net, chi-square test and Pearson correlation methods for the initial screening of stroke-related factors. Here, we consider using α=0.5 for the elastic net. Figure 3 shows that the impact of different parameters contained in these methods on the feature selection results. We can observe in Figures 3A, B the paths of regression coefficient changes based on Lasso and elastic net, with each curve corresponding to one feature variable. Figures 3C, D demonstrate the correlation of each feature with stroke. It is worth noting that we tend to select features with higher scores and p≤0.05 in the chi-square test (Pandis, 2016). According to Figure 3, Lasso, elastic net, chi-square test and Pearson correlation methods select 13, 15, 8 and 12 features, respectively.

**FIGURE 4:**
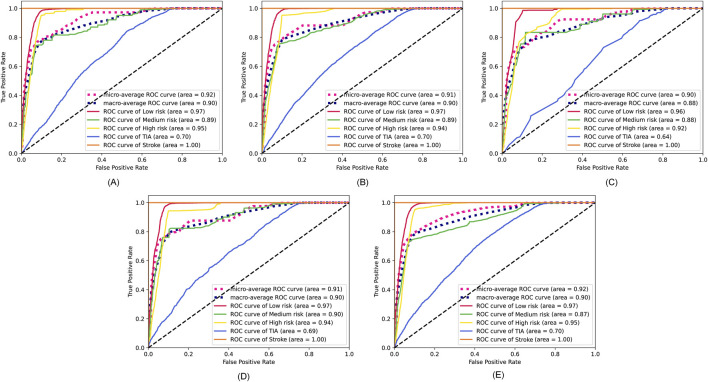
ROC curves corresponding to the feature sets selected by the four methods and the candidate feature set. **(A)** Lasso. **(B)** Elastic net. **(C)** Chi-square test. **(D)** Pearson correlation. **(E)** The robust candidate feature set.

The specific feature selection results of each method are shown in Table 4. Subsequently, we took the union of features selected by the four methods as the robust candidate feature set, which includes 16 features, i.e., AG, Gender, Smoking, MS, Occupation, ES, HS, HYP, AF, LDL-C, Diabetes, LE, Overweight, FHS/HYP/CHD, PLT, and PLH. The receiver operating characteristic (ROC) curves (Fan et al., 2006) corresponding to different feature sets are shown in Figure 4. We find that using the candidate feature set achieves better prediction results than features selected by individual methods. It illustrates that the first step of feature selection is of great significance for stroke risk diagnosis.

**TABLE 4:** Feature selection results of four methods.

Methods	Features
Lasso	AG, Gender, Occupation, HS, HYP, AF, Smoking, LDL-C, Diabetes, LE, Overweight, FHS/HYP/CHD
Elastic net	AG, Gender, Occupation, ES, HS, HYP, AF, Smoking, LDL-C, Diabetes, LE, Overweight, FHS/HYP/CHD, PLT, PLH
Chi-square Test	HS, HYP, AF, LDL-C, Diabetes, LE, Overweight, FHS/HYP/CHD
Pearson correlation	AG, ES, HS, HYP, AF, Smoking, LDL-C, Diabetes, LE, Overweight, FHS/HYP/CHD, MS

**FIGURE 5:**
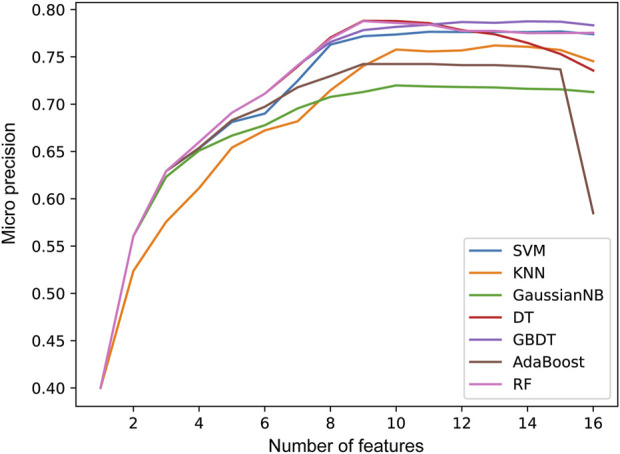
The prediction performance micro precision in seven machine learning methods with different number of features.

In the second step of feature selection, we eliminate risk factors with strong correlation between features. Based on the results of the first step of feature selection, we iterate through all candidate feature combinations. All feature combinations are evaluated under different machine learning methods as classifiers. The optimal feature combinations for different number of features are determined with respect to the evaluation results. Figure 5 shows the performance of the method with different numbers of feature variables. We see that as the number of features increases, the micro precision of most machine learning methods gradually improves and tends to stabilize. However, the performance of the DT and AdaBoost methods decreases significantly when the number of features is 9 and 15 respectively. When the number of features reaches 12, all seven machine learning methods overall achieve the best performance. Finally, we obtained risk factors that are highly relevant to stroke patients and have no redundant information among features, including Smoking, Occupation, ES, HS, HYP, AF, LDL-C, Diabetes, LE, Overweight, FHS/HYP/CHD, and PLT. It is worth noting that traditional methods consider age and gender to be strongly correlated with stroke risk (Howard et al., 2023; Ospel et al., 2023). However, two-level feature selection has removed them due to their redundancy with occupation and other risk factors. In contrast, the PLT features reflecting the climate of the patient’s location are preserved, and it has been confirmed that low temperatures are associated with an increased risk of stroke (Chen et al., 2013). This indicates that SRPNet could provide new insights for future risk screening.

### 3.2 Stroke risk prediction results

In this section, we validate the effectiveness of the SRPNet model on the CSDC dataset. Decision tree C5.0 (C5.0) (Ahmadi et al., 2018), random forests (RF) (Breiman, 2001),FCN, one-dimensional convolutional neural network (CNN), long short-term memory network (LSTM) and Transformer are used as comparison methods to predict stroke risk. Table 5 shows the prediction performance of the seven methods on the original CSDC data (all features) and the data after two-level feature selection (selected features). We can find that SRPNet model obtains the best prediction results in terms of the four evaluation metrics. The performance of all predictors after two-level feature selection is significantly better than their performance when using all features. This demonstrates that the two-level feature selection can effectively filter weak and redundant information, thus improving the results of all predictors. On the selected feature data, SRPNet outperformes FCN and Transformer by approximately 1.4%, 1.4%, 12% and 3.2% on metrics micro F1-score, micro precision, macro precision, Cohen’s Kappa coefficient. This reflects that deep fusion network can better explore potential relationships between risk factors. In summary, the proposed SRPNet model is reasonable and effective for predicting stroke risk.

**TABLE 5:** Comparison of stroke risk prediction results for the seven methods.

Methods	Selected features				All features			
	Micro F1-score	Micro precision	Macro precision	Cohen’s Kappa coefficient	Micro F1-score	Micro precision	Macro precision	Cohen’s Kappa coefficient
C5.0	0.9470	0.9470	0.7369	0.8828	0.9149	0.9149	0.7288	0.8068
RF	0.9478	0.9478	0.7672	0.8853	0.9167	0.9167	0.7170	0.8172
FCN	0.9257	0.9257	0.7119	0.8335	0.8906	0.8906	0.6811	0.7449
CNN	0.9421	0.9422	0.7316	0.8716	0.9371	0.9371	0.7310	0.8591
LSTM	0.9424	0.9424	0.8144	0.8723	0.9399	0.9399	0.7328	0.8654
Transformer	0.9480	0.9480	0.7449	0.8846	0.9198	0.9198	0.7396	0.8176
SRPNet	**0.9618**	**0.9618**	**0.8642**	**0.9165**	**0.9511**	**0.9511**	**0.8126**	**0.8920**

Note: The best experimental results are highlighted in bold.

Furthermore, to make the results more convincing, we evaluated six predictors on in-house data from affiliated hospital of Jining Medical University. The experimental results are recorded in Table 6. We can draw the similar conclusion that the proposed SRPNet model is an ideal and effective prediction tool of stroke risk. To explore the features that play a dominant role in precise classification, we removed each feature and obtained the prediction results for stroke risk. We found that after removing the hypertension (HYP) feature resulted in micro F1-score, micro precision, macro precision, and Cohen’s Kappa coefficient of 0.7, 0.7, 0.83, and 0.28 respectively, which had the greatest impact on stroke prediction performance. Secondly, gender and age also significantly influenced stroke classification, while they are identified as redundant features in the CSDC dataset. The reason is that the analysis conducted on the CSDC dataset involves complex stroke risk prediction, focusing on differences between multiple risk levels, whereas the in-house dataset only focuses on whether someone has a stroke, conducting a simple stroke prediction analysis. Understanding these risk factors can assist doctors in making quick and accurate stroke diagnoses.

**TABLE 6:** Prediction results based on our in-house dataset.

Methods	Micro F1-score	Micro precision	Macro precision	Cohen’s Kappa coefficient
C5.0	0.9000	0.9000	0.8750	0.7826
RF	0.9000	0.9000	0.9285	0.7826
FCN	0.9953	0.9953	0.9933	0.9917
CNN	0.8000	0.8000	0.8571	0.6000
LSTM	0.8000	0.8000	0.8000	0.6000
Transformer	0.9000	0.9000	0.9283	0.7826
SRPNet	**0.9978**	**0.9978**	**0.9954**	**0.9929**

Note: The best experimental results are highlighted in bold.

**FIGURE 6:**
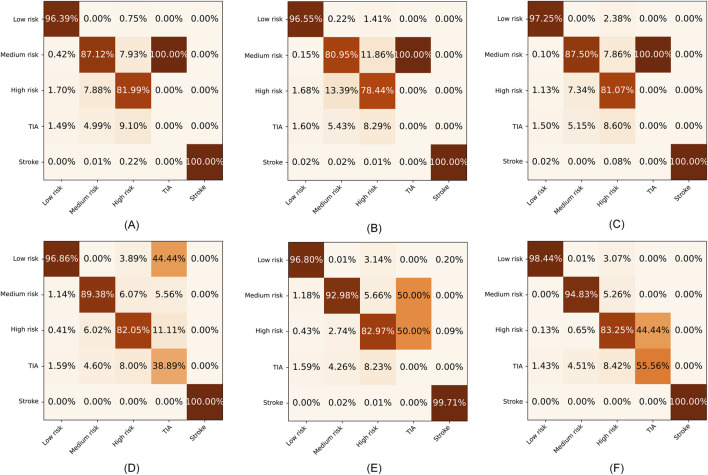
The confusion matrices for the six methods on the CSDC dataset. **(A)** C5.0. **(B)** FCN. **(C)** CNN. **(D)** LSTM. **(E)** Transformer. **(F)** SRPNet.

**FIGURE 7:**
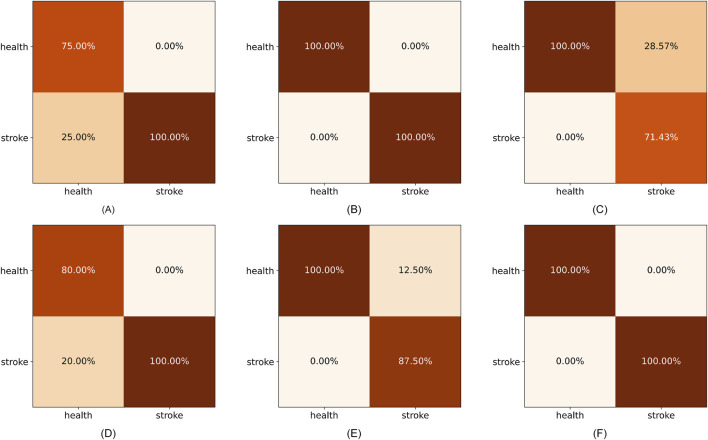
The confusion matrices for the six methods on the in-house dataset. **(A)** C5.0. **(B)** FCN. **(C)** CNN. **(D)** LSTM. **(E)** Transformer. **(F)** SRPNet.

To further evaluate the superiority of SRPNet, we visualize the confusion matrices obtained by the six methods on the CSDC dataset and the in-house dataset in Figures 6, 7, where the columns and rows are the predicted labels and true labels, respectively. It shows that compared to other methods, The SRPNet method wins in all categories in terms of prediction accuracy. Additionally, we discover that the history of stroke (HS) feature and the hypertension (HYP) feature significantly enhance the ability of almost all algorithms in Figure 6 to detect stroke effectively.

## 4 Conclusion

In this paper, a novel prediction model based on two-level feature selection and deep fusion network is proposed for stroke risk prediction. Compared with traditional feature selection methods, the proposed two-level feature selection method not only focuses on the importance of individual these features, but also eliminates redundant information among important features. Furthermore, the proposed deep fusion network harnesses Transformer and fully connected networks to capture feature dependencies and model the non-linear relationships among features, respectively. Experimental results on the CSDC database and in-house dataset demonstrate that our proposed prediction model outperforms other representative methods. This prediction model can rapidly identify high-quality stroke risk factors and improve the accuracy of stroke prediction for patients, thereby effectively assisting doctors in formulating rational diagnosis and treatment plans.

The features included in the CSDC database and in-house dataset are limited. In the future, we will collect more clinical indicator features related to stroke for model training and testing. And we will also work on applying the proposed model to predict other diseases, demonstrating its generalizability. It's worth noting that researchers have the flexibility to substitute the feature selection method used in SRPNet with other methods that are frequently applied in the context of medical information, tailored to their specific requirements.

## Data Availability

The datasets presented in this study can be found in online repositories. The names of the repository/repositories and accession number(s) can be found below: https://github.com/zhangdaoliang/SRPNet.
